# Aetiology of Acute Respiratory Insufficiency in Patients With Ischaemic Stroke Studied by Chest CT Scan

**DOI:** 10.1111/ene.70125

**Published:** 2025-03-25

**Authors:** Omid Shirvani, Patricia Fischbein, Zeynep Bendella, Piergiorgio Profico, Franziska Dorn, Gabor C. Petzold, Sebastian Stösser

**Affiliations:** ^1^ Department of Vascular Neurology University Hospital Bonn Bonn Germany; ^2^ German Center for Neurodegenerative Diseases Bonn Germany; ^3^ Department of Diagnostic and Interventional Neuroradiology University Hospital Bonn Bonn Germany

**Keywords:** acute pulmonary condition, acute respiratory insufficiency, chest CT scan, stroke, stroke complication

## Abstract

**Background:**

Acute respiratory insufficiency (ARI) is considered a serious life‐threatening complication after ischaemic stroke. The aim of this study was to identify the most common aetiologies of ARI after stroke and their association with patients' outcome.

**Methods:**

This retrospective study was conducted at the University Hospital Bonn, involving patients with acute ischaemic stroke who underwent chest CT scans for ARI between 2017 and 2022. We collected clinical and demographic data, laboratory parameters, vital signs, as well as outcome parameters. CT scans were reviewed by a radiologist. The dataset was analysed to identify the most frequent aetiologies and their associations to outcome parameters.

**Results:**

We included 236 patients with a median age of 75 years and a median NIHSS score of 11. In‐hospital mortality accounted for 30.5%. The most frequent pulmonary conditions on CT, in order of prevalence, included bronchitis/bronchiolitis (66.1%), atelectasis (66.1%), pleural effusion (60.6%), pneumonia (53%), pulmonary oedema (37.3%), and pulmonary artery embolism (27.5%). Bronchitis/bronchiolitis was an independent risk factor for mortality (OR = 3.17, 95% CI: 1.11–8.79, *p* = 0.03). A higher number of pulmonary conditions decreased the likelihood of discharge to home, and non‐survivors had worse vital/laboratory parameters.

**Conclusions:**

We identified six key pulmonary aetiologies of ARI after ischaemic stroke, with bronchitis/bronchiolitis notably linked to in‐hospital mortality in our study cohort. An increased number of these acute pulmonary conditions decreased the likelihood of discharge to home. Early chest CT/CT‐angiography may help to identify patients at high risk for in‐hospital mortality and to initiate appropriate treatment early.

## 1 Introduction

Ischaemic stroke ranks among the most threatening medical emergencies, severely affecting patients' quality of life and mortality rates [1]. Stroke not only poses an immediate threat due to brain injury, but also leads to a wide range of secondary complications beyond the nervous systems [2, 3]. In particular, the development of acute respiratory conditions such as pneumonia plays a crucial role in post stroke management [3]. The pathogenesis of acute respiratory insufficiency (ARI) in stroke patients is heterogeneous, covering immobility, insufficient coughing, dysphagia, neural lung alterations, and infections due to stoke‐induced immunosuppression [4, 5]. Early diagnostics and appropriate therapies are vital for reducing mortality and shortening hospital lengths. The Pneumonia In Stroke ConsEnsuS (PISCES) group has thus established operational criteria to clearly define the diagnosis of stroke‐associated pneumonia (SAP) [6]. Prophylactic measures including posture correction, early dysphagia screening, and certain medications may help mitigate the incidence of respiratory complications [7, 8, 9]. In contrast, antibiotic therapy should be reserved solely for confirmed bacterial infections, as preventive antibiotic use has proven ineffective [10]. The interactions within the “heart‐lung axis” continue to be a crucial focus of ongoing research. The aim of this study was to identify the most common aetiologies of ARI after stroke and their association to patients' outcomes. Understanding this relationship is essential for advancing comprehensive post‐stroke management strategies.

## 2 Patients and Methods

Our study was conducted at the Department of Vascular Neurology, University Hospital Bonn, with a monocentric, retrospective design. We included patients with acute ischaemic stroke who underwent a chest CT scan for ARI admitted to our hospital from 2017 to 2022. Patients with acute ischaemic stroke and chest CT scans were identified by querying the ICD‐10 code I63 for cerebral infarction and the OPS‐301 code 3–202 for native computed tomography of the thorax as well as the OPS‐301 code 3–222 for computed tomography of the thorax with contrast medium. ARI was identified by specific keywords in CT requests, such as “pneumonia” or “acute respiratory failure”. Patients were excluded from the study if chest CT scans were ordered for other reasons, such as cancer or cardiac disease. Additionally, patients with inaccurate medical records (e.g., missing CT scans) were also excluded. We recorded clinical/demographic parameters such as pre‐morbidities, findings from plain chest radiographs (CXR), stroke characteristics, acute stroke treatment, and discharge information from the medical records. This review was performed by two medical students and validated by two experienced medical/scientific supervisors. CXRs were only taken into consideration if they were recorded ±1 day around the chest CT scan. The pre‐existing morbidities covered arterial hypertension, diabetes mellitus, dyslipidaemia, known cardiac diseases, and known pulmonary diseases. Pulmonary diseases encompassed a range of conditions, such as asthma, chronic obstructive pulmonary disease, and chronic bronchitis. Cardiac diseases included conditions such as chronic heart failure, coronary artery disease, and valvular diseases. Additionally, we documented smoking history as an additional cardiovascular risk factor. Dysphagia was classified based on a comprehensive swallowing assessment by speech therapists as follows: severe dysphagia (with no oral intake), moderate to severe dysphagia (with intake of thick liquidised food and no intake of fluids), moderate dysphagia (with intake of pureed food and thickened liquids), and mild dysphagia (with soft food und regular fluid intake). The most recent swallowing assessment prior to the CT scan was taken into account. Furthermore, we collected laboratory parameters and vital signs (worst value) from the day of the CT scan for ARI. Details on the collected data are provided in Table S1.

All patients underwent multislice‐CT imaging of the thorax in supine positioning with or without administration of iodinated contrast on a clinical CT scanner (IQon Spectral CT, Philips Healthcare). Typical imaging parameters were: slice thickness 0.625 mm, tube voltage 120 kVp, and tube current (exposure time product) 30 mAs. Image datasets were retrieved from the institutional picture archiving and communication system (IMPAX, Agfa Healthcare, Belgium). All CT scans were reviewed by a board‐certified radiologist who was blinded to acquired data, in particular to stroke‐related characteristics and outcome parameters. In the first step, a diagnosis was assigned to the given chest CT scan based on the following criteria, which were answered with yes or no: lobar pneumonia, bronchopneumonia, bronchitis/bronchiolitis, pleural effusion, atelectasis, pulmonary embolism (PE), and others. In the next step, the respective diagnoses were quantified: pneumonia was classified using a score for the extent of changes in six lung segments. A score ranging from 0 to 4 (0: no involvement; 1: 1%–25% involvement; 2: 26%–50%; 3: 51%–75%; 4: 76%–100%) was assigned to each lung zone, and the scores were summed to provide a total score ranging from 0 to 24. Additionally, for pneumonia, the type of changes was specified, with the following options: consolidation, ground‐glass opacity, centrilobular nodules, and bronchial wall thickening. The pleural effusion was volumetrically assessed using a calculator provided by Hazlinger et al. [11]. Atelectasis was classified according to the following criteria: complete lung, multilobar, lobar, or segmental. PE was categorized as central, lobar, segmental, or subsegmental, and quantified using the Qanadli index. The Qanadli index assesses obstruction (partial or complete) as well as downstream perfusion in 10 lung segments, with values ranging from 0 to 40 [12]. Additionally, incidental findings were also recorded.

The study was conducted in accordance with the Declaration of Helsinki. The study protocol was approved by the Ethics Committee of the Faculty of Medicine Bonn, BV181/2022. Informed consent was not required from the participants or their legal guardians. The confidentiality of patient data was maintained throughout the study.

## 3 Statistics

For statistical analysis, we employed Python (version 3.11.5) and SPSS. ChatGPT‐4 (OpenAI) provided assistance with coding and language editing. Our analyses, including the association of acute pulmonary conditions with health‐related parameters such as functional outcome or hospitalisation length, were investigated in an exploratory manner. Statistical significance was set at *p* < 0.05. Data that were not normally distributed were presented using the median and the first and third quartiles, while normally distributed data were described by mean and standard deviation. Normality of distribution was assessed using histograms and the Shapiro–Wilk test. We utilised a two‐tailed Mann–Whitney *U*‐test, a two‐tailed independent *t*‐test, and a chi‐square test of independence to investigate differences in data distribution between two groups. Spearman's correlation coefficient was used for correlation analyses. Linear, ordinal, and logistic regression analyses were conducted to investigate independent associations between acute pulmonary conditions and outcome parameters. Covariates were added by clinical reasoning. Details on regression analyses (covariates, beta coefficients, odds ratios, and p‐values) are provided in the Supplementals. Missing data was not imputed. The number of missing values is detailed in Table S2.

## 4 Results

**FIGURE 1:**
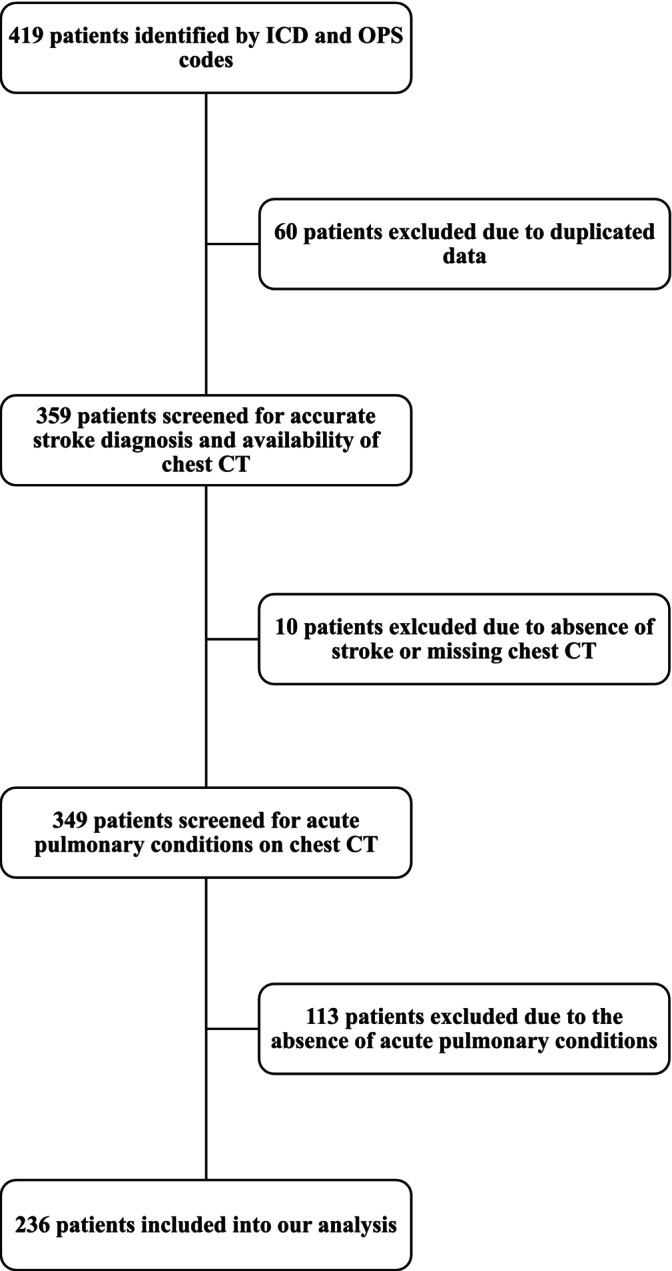
Flow chart for inclusion and exclusion of patients identified by ICD and OPS codes. CT = computed tomography, ICD = International Statistical Classification of Diseases and Related Health Problems (used ICD code: ICD‐10 I63 = cerebral infarction), OPS = Operations‐ and Procedures‐key (used OPS codes: OPS‐301 3–202 = native computed tomography of the thorax, OPS‐301 3–222 = computed tomography of the thorax with contrast medium).

We screened 419 ischaemic stroke patients who underwent a chest CT and included 236 patients with a median age of 75 years and a median NIHSS score of 11 at admission (Figure 1, Table 1). Almost half of the study population was female (45.7%, *n* = 108). Most patients had an independent functional state before hospital admission (median: 0, Q1‐Q3: 0–2). Regarding acute stroke therapy, 20.9% (*n* = 46) received intravenous thrombolysis, while 34.1% (*n* = 75) underwent mechanical thrombectomy. The majority of patients (61.4%, *n* = 145) were treated on an intensive care unit. Cardioembolism (37.2%, *n* = 88) was the most common aetiology of stroke. At discharge, surviving patients had a median mRS of 4. In‐hospital mortality accounted for 30.5% (*n* = 72) of all patients. 12.7% (*n* = 30) were able to be discharged at home after a median stay of two weeks. Non‐survivors were significantly older (78 vs. 73 years, *p* < 0.01) and had significantly shorter hospitalisation lengths (10.5 vs. 15.5 days, *p* < 0.01) compared to survivors. Furthermore, non‐survivors were more likely to have pre‐existing cardiac conditions, experienced more severe dysphagia, and required a higher level of medical care (Table 1). Among the recorded pre‐morbidities, arterial hypertension was the most prevalent, affecting 85% (*n* = 187) of all patients, followed by cardiac diseases (71.3%, *n* = 154), dyslipidaemia (58.2%, *n* = 121), diabetes mellitus (39.2%, *n* = 82), and pulmonary diseases (32.9%, *n* = 70).

**TABLE 1:** Demographic and clinical characteristics of patients with ischaemic stroke and acute respiratory insufficiency.

Parameter	All patients (*n* = 236)	Survived (*n* = 164)	Deceased (*n* = 72)
Age, years, median (Q1‐Q3)	75 (62–82)	73 (61–80.3)	78 (68–83)*
Sex, female, *n*/*N* (%)	108/236 (45.7%)	71/164 (43.3%)	37/72 (51.4%)
Arterial hypertension, *n*/*N* (%)	187/220 (85%)	127/154 (82.5%)	60/66 (90.9%)
Diabetes mellitus, *n*/*N* (%)	82/209 (39.2%)	61/152 (40.1%)	21/57 (36.8%)
Dyslipidaemia, *n*/*N* (%)	121/208 (58.2%)	89/156 (57.1%)	32/52 (61.5%)
Known cardiac disease, *n*/*N* (%)	154/216 (71.3%)	104/155 (67.1%)	50/61 (82%)*
Known pulmonary disease, *n*/*N* (%)	70/213 (32.9%)	54/155 (34.8%)	16/58 (27.6%)
Active smoker, *n*/*N* (%)	60/199 (30.2%)	44/147 (29.9%)	16/52 (30.8%)
Premorbid mRS, median (Q1‐Q3)	0 (0–2)	0 (0–2)	1 (0–2.5)
NIHSS on admission, median (Q1‐Q3)	11 (5–17)	8 (4–16)	14 (9–20)
Intravenous thrombolysis, *n*/*N* (%)	46/220 (20.9%)	35/154 (22.7%)	11/66 (16.7%)
Endovascular thrombectomy, *n*/*N* (%)	75/220 (34.1%)	52/154 (33.8%)	23/66 (34.8%)
TOAST, *n*/*N* (%)	*No significant difference across all aetiologies*		
Stroke of undetermined aetiology	67/236 (28.3%)	45/164 (27.4%)	22/72 (30.6%)
Stroke with other determined aetiology	30/236 (12.7%)	24/164 (14.6%)	6/72 (8.3%)
Small‐vessel occlusion	15/236 (6.3%)	11/164 (6.7%)	4/72 (5.56%)
Cardioembolism	88/236 (37.2%)	62/164 (37.8%)	26/72 (36.1%)
Large‐artery atherosclerosis	36/236 (15.2%)	22/164 (13.4%)	14/72 (19.4%)
Severity of dysphagia, *n*/*N* (%)	*No significant difference across all severities*		
Severe dysphagia	132/227 (58.1%)	76/159 (47.8%)	56/68 (82.4%)*
Moderate to severe dysphagia	19/227 (8.4%)	14/159 (8.8%)	5/68 (7.4%)
Moderate dysphagia	10/227 (4.4%)	9/159 (5.7%)	1/68 (1.5%)
Mild dysphagia	19/227 (8.4%)	16/159 (10.1%)	3/68 (4.4%)
No dysphagia	47/227 (20.7%)	44/159 (27.7%)	3/68 (4.4%)*
mRS at discharge, median (Q1‐Q3)	5 (4–6)	4 (3–5)	6 (6–6)*
NIHSS at discharge, median (Q1‐Q3)	6.5 (2–12.8)	6.5 (2–12.8)	—
Exitus letalis during hospitalisation, *n*/*N* (%)	72/236 (30.5%)	0/164 (0%)	72/72 (100%)*
Duration of neurological hospital stay, days, median (Q1‐Q3)	14 (7–22.3)	15.5 (9–25)	10.5 (5–16.3)*
Discharge mode, *n*/*N* (%)	*Significant difference across all categories*		
Discharge to home	30/236 (12.7%)	30/164 (18.3%)	0/72 (0%)*
Rehabilitation	53/236 (22.4%)	53/164 (32.3%)	0/72 (0%)*
Transfer to another ward or hospital	81/236 (34.3%)	81/164 (49.4%)	0/72 (0%)*
No discharge due to Exitus letalis	72/236 (30.5%)	0/164 (0%)	72/72 (100%)*
Barthel scale, median (Q1‐Q3)	12.5 (0–40)	12.5 (0–40)	12.5 (0–31.3)
Highest level of care, *n*/*N* (%)	*Significant difference across all categories*		
Intensive Care Unit	145/236 (61.4%)	91/164 (55.5%)	54/72 (75%)*
Stroke Unit	87/236 (33%)	72/164 (43.9%)	15/72 (20.8%)*
General ward	4/236 (1.6%)	1/164 (0.6%)	3/72 (4.2%)

Abbreviations: mRS = Modified Ranking Scale, *n* = number of patients, *N* = number of available data, NIHSS = National Institutes of Health Stroke Scale, Q1 = first quartile, Q3 = third quartile, SD = standard deviation, TOAST = Trial of Org 10,172 in Acute Stroke Treatment.

* Indicates statistically significant differences between both subgroups Deceased and Survived; Mann–Whitney *U*‐Test and chi‐square test of independence applied.

**FIGURE 2:**
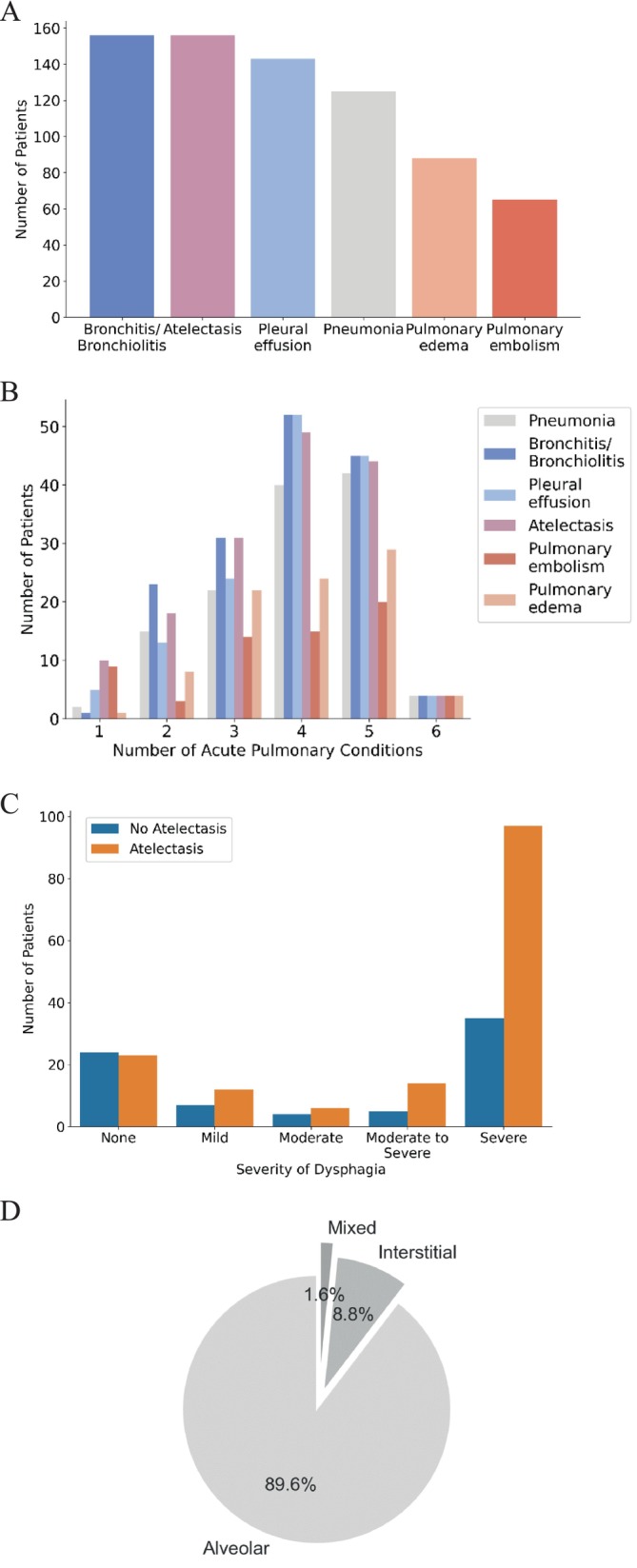
(A) Prevalence of acute pulmonary conditions in acute respiratory insufficiency. (B) Contribution of acute pulmonary conditions to multiple aetiologies of acute respiratory insufficiency. (C) Association between the severity of dysphagia and the presence of atelectasis. (D) Type of pneumonia among patients with pneumonia.

**FIGURE 3:**
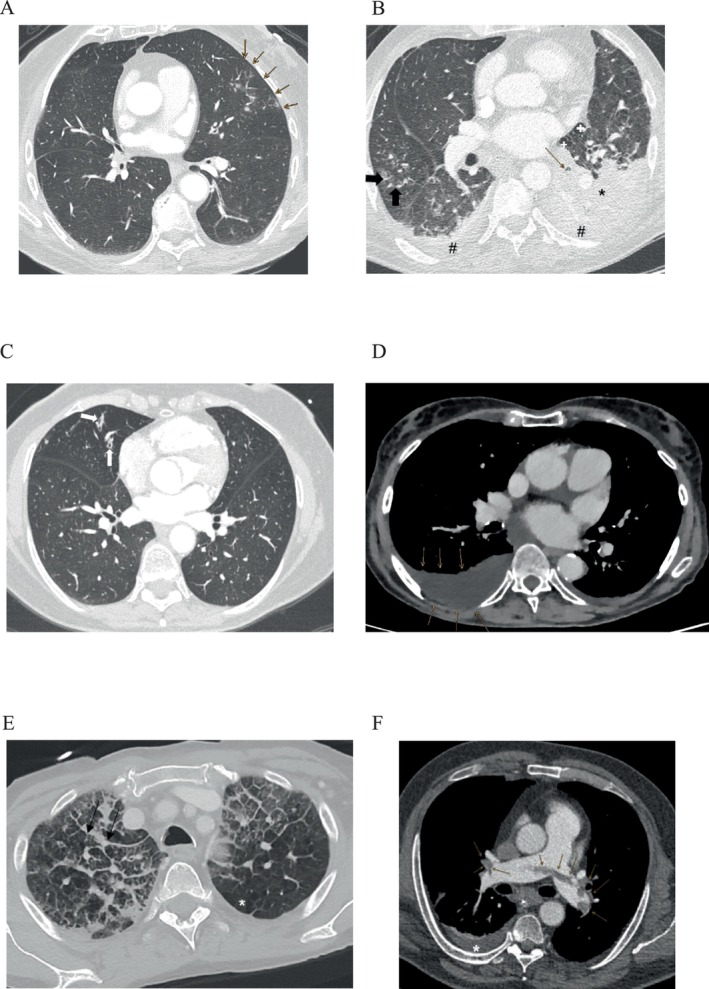
(A) Axial chest CT scan with lung window reconstruction. Arrows indicate areas of patchy consolidations and ground‐glass opacities in the lateral aspect of the left upper lobe, consistent with pneumonia. (B) Axial chest CT scan with lung window. A left lower lobe atelectasis is present due to obstruction of the bronchus (thin arrow), likely by mucus. Adjacent dystelectasis is visible in the left upper lobe (*). Emphysematous bullae are also observed in the left upper lobe (+). Additionally, bilateral pleural effusions are also present (#). Pneumonic infiltrates are noted in the right middle and lower lobes (thick arrow). (C) Axial chest CT scan with lung window reconstruction. Thickening of individual bronchial walls with partial mucus impaction is observed in the middle lobe, consistent with bronchitis/bronchiolitis (white arrows). (D) Axial chest CT scan with soft tissue window. Arrows highlight a right‐sided pleural effusion. (E) Axial chest CT scan with lung window reconstruction. A predominant interstitial oedema is observed, with adjacent centrilobular opacities indicative of pulmonary venous congestion and scattered alveolar congestion infiltrates. Centrilobular emphysema is present (*). Additionally, pleural apical thickening is visible (arrows). (F) Axial chest CT scan with soft tissue window. A central pulmonary embolism is present, with extensive thrombotic material bilaterally extending into the peripheral pulmonary arteries (arrows). A right‐sided pleural effusion (*) is also present.

Investigating the chest CT scans, we found that the most prevalent pulmonary conditions, in descending order, were bronchitis/bronchiolitis (66.1%, *n* = 156), atelectasis (66.1%, *n* = 155), pleural effusion (60.6%, *n* = 143), pneumonia (53%, *n* = 125), pulmonary oedema (37.3%, *n* = 85), and PE (27.5%, *n* = 62) (Figure 2A). Figure 3 shows representative chest CT images illustrating the most prevalent pulmonary conditions. The order of prevalence remained consistent between surviving and non‐surviving patients (Table S3). Most patients exhibited four to five acute pulmonary conditions, while the majority had at least three (Figure 2B). In 13 cases, the chest CT scan did not reveal an acute pulmonary condition as the cause of the ARI (Table S4). We further characterised the conditions in depth based on patients' CT scans (Table 2). About 20.3% (*n* = 48) of our patients had atelectasis on a multilobar level, while segmental atelectasis with 35.2% (*n* = 83) occurred the most often (Table 2). The severity of dysphagia was significantly associated with the likelihood of developing atelectasis (Chi‐square test of independence, *p* = 0.04) (Figure 2C). Regarding patients with pneumonia, 89.6% (*n* = 112) of affected patients exhibited alveolar pneumonia, 8.8% (*n* = 11) showed interstitial pneumonia, and 1.6% (*n* = 2) had a mixed type (Figure 2D). Among those with alveolar or mixed pneumonia, 7% (*n* = 9) demonstrated lobar pneumonia, while the vast majority, 93% (*n* = 109), had bronchopneumonia (Figure S1A). Investigating the CT scores for pneumonia, we neither found a significant difference between survivors and non‐survivors, nor a correlation with the mRS at discharge (Figure S1B,C). Similarly, there were no such associations concerning the volume of pleural effusion and the Qanadli Index for PE (Figure S2). However, non‐survivors were more likely to develop alveolar pulmonary oedema in comparison to survivors (Table 2).

**TABLE 2:** Radiological characteristics of acute pulmonary conditions.

Parameter	All patients (*n* = 236)	Survived (*n* = 164)	Deceased (*n* = 72)
Pneumonia, *n*/*N* (%)	*No significant difference across all categories*		
Alveolar	112/236 (47.5%)	80/164 (48.8%)	32/72 (44.4%)
Interstitial	11/236 (4.7%)	7/164 (4.3%)	4/72 (5.6%)
Mixed	2/236 (0.9%)	2/164 (1.2%)	0/72 (0%)
No pneumonia	111/236 (47%)	75/164 (45.7%)	36/72 (50%)
Type of alveolar/mixed pneumonia, *n*/*N* (%)	*No significant difference across all categories*		
Lobar pneumonia	8/114 (7%)	6/82 (7.3%)	1/32 (3.1%)
Bronchopneumonia	106/114 (93%)	76/82 (92.7%)	31/32 (96.9%)
CT score for pneumonia, median (Q1‐Q3)	8 (4–12)	7 (4–12)	9 (3–13)
Pneumonia with ground glass opacity, *n*/*N* (%)	165/236 (69.9%)	113/164 (68.9%)	52/72 (72.2%)
Pneumonia with centrilobular nodules, *n*/*N* (%)	118/236 (50%)	80/164 (48.8%)	38/72 (52.8%)
Pneumonia with bronchial wall thickening, *n*/*N* (%)	158/236 (66.9%)	107/164 (65.2%)	51/72 (70.8%)
Pleural effusion, *n*/*N* (%)	143/236 (60.6%)	98/164 (59.8%)	45/72 (62.5%)
Volume of pleural effusion, ml, median (Q1‐Q3)	281.1 (112.4–575.43)	288.3 (142.2–564.8)	230.4 (82.3–586)
Bronchitis/Bronchiolitis, *n*/*N* (%)	156/236 (66.1%)	104/164 (63.4%)	52/72 (72.2%)
Atelectasis, *n*/*N* (%)	*No significant difference across all categories*		
Whole lung	3/236 (1.3%)	1/164 (0.6%)	2/72 (2.8%)
Multilobar	48/236 (20.3%)	33/164 (20.1%)	15/72 (20.8%)
Lobar	21/236 (8.9%)	15/164 (9.1%)	6/72 (8.3%)
Segmental	83/236 (35.2%)	59/164 (36%)	24/72 (33.3%)
No atelectasis	81/236 (34.3%)	56/164 (34.1%)	25/72 (34.7%)
Localisation of pulmonary artery embolism, *n*/*N* (%)	*No significant difference across all categories*		
Central	21/236 (8.9%)	12/164 (7.3%)	9/72 (12.5%)
Lobar	6/236 (2.5%)	3/164 (1.8%)	3/72 (4.2%)
Segmental	17/236 (7.2%)	13/164 (7.9%)	4/72 (5.6%)
Subsegmental	18/236 (7.6%)	15/164 (9.1%)	3/72 (4.2%)
No pulmonary artery embolism	174/236 (73.7%)	121/164 (73.8%)	53/72 (73.6%)
Qanadli Index for pulmonary artery embolism, median (Q1‐Q3)	25 (11.88–63.13)	25 (10–62.5)	45 (15–70)
Type of pulmonary oedema, *n*/*N* (%)	*Significant difference across all categories*		
Interstitial	75/236 (31.8%)	57/164 (34.8%)	18/72 (25%)
Alveolar	10/236 (4.2%)	3/164 (1.8%)	7/72 (9.7%)*
No oedema	151/236 (64%)	104/164 (63.4%)	47/72 (65.3%)

Abbreviations: CT = computed tomography, *n* = number of patients, *N* = number of available data, Q1 = first quartile, Q3 = third quartile.

* Indicates statistically significant differences between both subgroups Exitus letalis and Survived; Mann–Whitney *U*‐Test and chi‐square test of independence applied.

**FIGURE 4:**
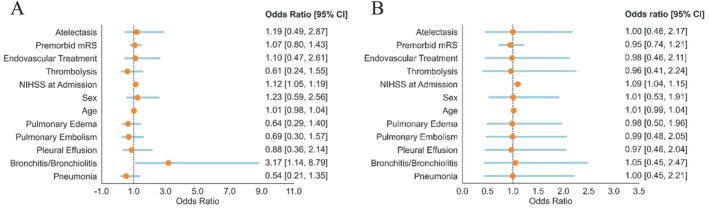
(A) Logistic regression analysis for in‐hospital mortality. (B) Ordinal regression analysis for modified Ranking Scale (mRS) shift.

Furthermore, we conducted regression analyses to determine independent associations between acute pulmonary conditions and two outcomes: in‐hospital mortality and mRS shift. The NIHSS score at admission (OR = 1.12, *p* < 0.01), along with bronchitis/bronchiolitis (OR = 3.17, *p* = 0.03) were both independent risk factors for in‐hospital mortality (Figure 4A, Table S5). However, only the NIHSS score at admission (OR = 1.09, *p* < 0.01) was identified as an independent risk factor for the mRS shift (Figure 4B, Table S6). The sum of acute pulmonary conditions was not associated with either outcome (Tables S7 and S8).

Moreover, we conducted regression analyses to examine the associations between acute pulmonary conditions and two further outcomes: the length of hospitalisation and the likelihood of discharge to home. Neither the number of acute pulmonary conditions nor the specific conditions themselves were associated with the duration of the hospital stay (Tables S9 and S10). Regarding discharge to home, we found that having more acute pulmonary conditions reduced the chance of being discharged to home (OR = 0.56, *p* < 0.01), but no specific diagnosis showed a significant association (Tables S11 and S12).

**FIGURE 5:**
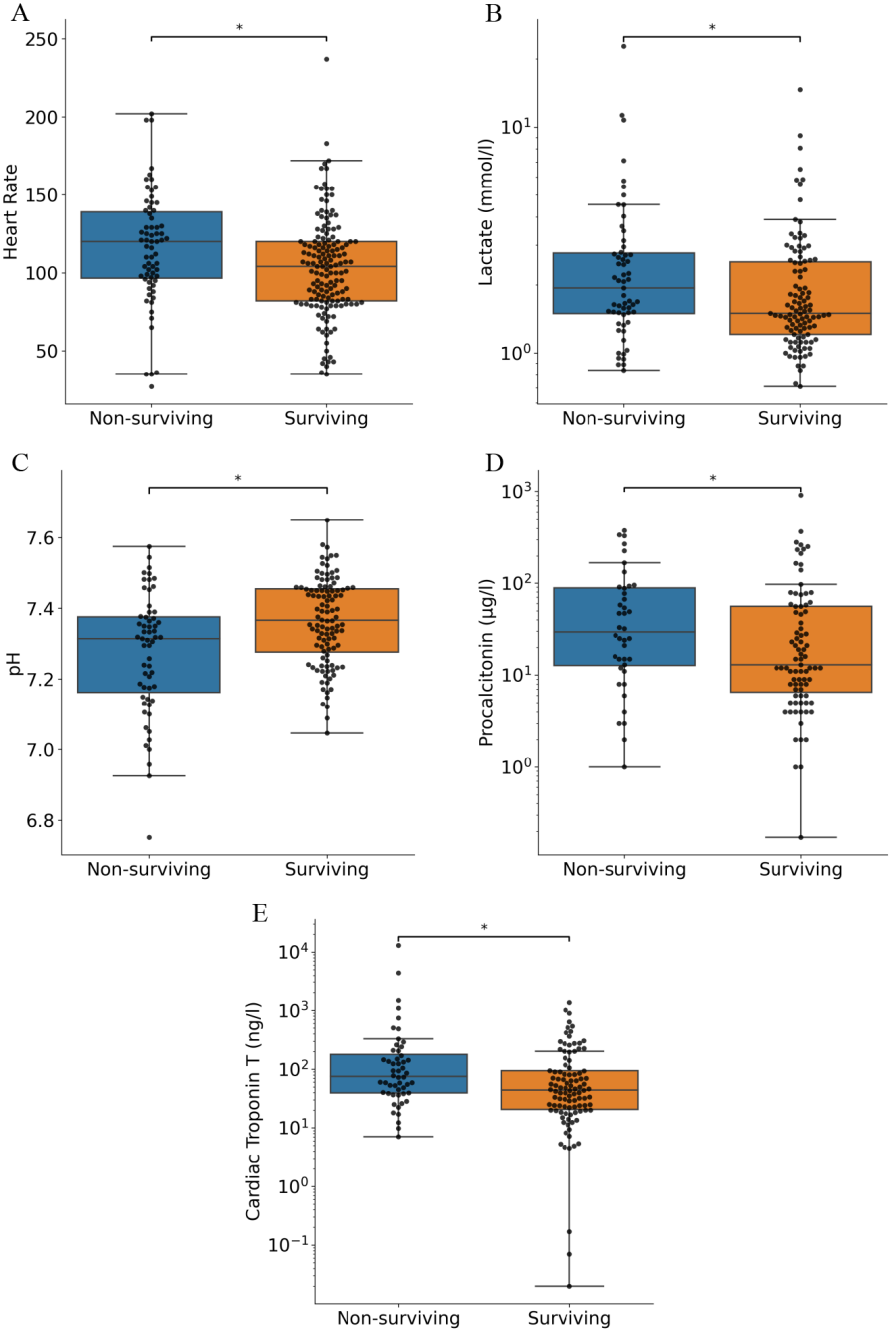
Biomarkers indicating more pronounced systemic inflammation and myocardial damage in non‐surviving patients at the day of respiratory failure. * Indicates statistically significant differences between both subgroups; Mann‐Whitney‐U‐Test and independent, unpaired t‐test applied.

We further investigated vital signs and laboratory parameters of patients with ischaemic stroke on the day of the chest CT scan (Table 3, Figure 5). The median recorded oxygen saturation of all patients was 89.5%. Notably, the group of non‐survivors exhibited significantly worse parameters compared to survivors (Table 3, Figure 5) including increased heart rates (116 vs. 103 beats/min, *p* = 0.01) and reduced pH in blood gas analyses (7.28 vs. 7.36, *p* < 0.01), accompanied by higher lactate levels (1.9 vs. 1.5 mmol/L, *p* = 0.03). Additionally, procalcitonin (0.5 vs. 0.2 ng/L, p = 0.03) and cardiac Troponin T (75 vs. 44 ng/L, *p* < 0.01) levels were higher. Taking all these signs into account, non‐surviving patients displayed more pronounced markers of ARI and systemic inflammation.

**TABLE 3:** Vital signs and laboratory parameter of patients with ischaemic stroke at the day of acute respiratory insufficiency.

Parameter	All patients (*n* = 236)	Survived (*n* = 164)	Deceased (*n* = 72)
Respiratory frequency, breath/min, median (Q1‐Q3)	28 (24–34)	28 (22–34)	27 (24–33)
Saturation of oxygen, %, median (Q1‐Q3)	89.5 (81.75–93)	90 (83–94)	88 (80–92)
Heart frequency, beats/min, mean (SD)	107.2 (33.5)	103.3 (31.7)	116 (36)*
Systolic blood pressure, mmHg, mean (SD)	161.4 (43.1)	162.8 (36.8)	158.3 (54.7)
Diastolic blood pressure, mmHg, mean (SD)	80.6 (33.9)	82.1 (31.1)	77.4 (39.3)
Mean arterial blood pressure, mmHg, median (Q1‐Q3)	111.4 (90–125.3)	106 (91.5–124.5)	104 (85–127)
Temperature, °C, mean (SD)	37.1 (1.4)	37.2 (1.3)	36.9 (1.6)
pH from blood gas analysis, mean (SD)	7.33 (0.15)	7.4 (0.12)	7.27 (0.17)*
Lactate from blood gas analysis, mmol/l, median (Q1‐Q3)	1.63 (1.29–2.64)	1.5 (1.21–2.53)	1.94 (1.5–2.77)*
Leukocytes, G/l, median (Q1‐Q3)	11.93 (8.98–15.66)	11.48 (8.81–15.22)	12.11 (9.36–16.29)
C‐reactive protein, mg/l, median (Q1‐Q3)	54.27 (19.3–96.52)	50.71 (18.04–89.63)	69.05 (29–134.79)
Cardiac troponin T, ng/l, median (Q1‐Q3)	52.9 (24.48–142.5)	44.35 (20.63–94.23)	75.15 (39.4–179)*
Procalcitonin, ng/l, median (Q1‐Q3)	0.23 (0.1–0.86)	0.2 (0.09–0.71)	0.47 (0.15–1.56)*

Abbreviations: G/l = giga per liter, mmHg = millimetre mercury, *n* = number of patients, Q1 = first quartile, Q3 = third quartile, SD = standard deviation.

* Indicates statistically significant differences between both subgroups Deceased and Survived; Mann–Whitney *U*‐Test and independent, unpaired *t*‐test applied.

Furthermore, we analysed CXRs obtained ±1 day around the chest CT scan for evidence of acute pulmonary conditions. Of the 236 patients in our study, 104 underwent a CXR within this period. The sensitivity of CXR to detect each pulmonary condition with CT findings as gold standard was as follows: 0% for bronchitis/bronchiolitis (0/78 cases), 49.4% for atelectasis (38/77), 70.1% for pleural effusion (47/67), 67.2% for pneumonia (41/61), 50% for pulmonary oedema (18/36), and 0% for PE (0/31).

## 5 Discussion

ARI represents a frequent and life‐threatening complication following ischaemic stroke [13, 14]. In our study, we analysed the aetiology of ARI in a cohort of over 200 stroke patients by chest CT scans, which are considered the gold standard diagnostic for ARI of suspected pulmonary aetiology [15].

We identified the primary aetiologies of ARI in descending order of frequency to be bronchitis/bronchiolitis, atelectasis, pleural effusion, pneumonia, pulmonary oedema, and PE among both surviving and non‐surviving patients. Bronchitis/bronchiolitis was not only among the two most prevalent acute pulmonary conditions, but also the only one that was independently associated with in‐hospital mortality. It remains unclear whether bronchitis/bronchiolitis was present prior to the stroke or developed subsequently in our cohort. Evidence suggests that chronic bronchitis may act as a risk factor for stroke [16]; of note, airway infections are recognised as a risk factor for ischaemic stroke in general [17]. However, a possible causal pathophysiological sequence might involve the development of bronchitis/bronchiolitis due to infection or chemical irritation from recurrent aspiration in dysphagic stroke patients, which could then –in case of infection‐even spread to the lung parenchyma per continuitatem and ultimately lead to pneumonia. This hypothesis is supported by our finding that most pneumonia patients presented with bronchopneumonia.

SAP is a frequent complication of stroke [13]. According to a meta‐analysis from 2018, approximately 8% of stroke patients develop SAP [18]. In our study population, we observed a higher incidence of pneumonia (53%), which is explained by our focus on patients with ARI. Most of our patients were treated in ICU, where spontaneous breathing is either assisted or replaced by mechanical ventilation in most patients. Mechanical ventilation is considered one of the major risk factors for SAP [19]. Numerous studies have explored prevention and treatment strategies for SAP. Of note, prophylactic antibiotics have been demonstrated to be ineffective [18]. In contrast, initiating appropriate antibiotics upon evidence of pneumonia has been recommended [10]. Additional preventive measures include modifying patient posture towards an upright position [8], conducting early dysphagia screening [9], and maintaining oral hygiene [20]. Regarding pharmacological interventions, evidence suggests that statins may offer protective benefits [7], whereas proton pump inhibitors could contribute to the development of SAPs [21].

Furthermore, atelectasis was one of the two most prevalent acute pulmonary conditions beside bronchitis/bronchiolitis. Atelectasis has various causes, including acute pulmonary conditions like those in stroke patients (e.g., SAP) as well as further stroke‐related risk factors such as immobility, reduced cough strength, and aspiration [22]. Our patients demonstrated an association between the severity of dysphagia and the presence of atelectasis, which is most likely a consequence of aspiration. Prophylactic and therapeutic measures include maintaining an upright position, regular postural changes, tracheal suctioning, positive pressure ventilation, and encouraging deep breaths [22, 23]. In cases where suctioning and coughing are unsuccessful, and a mechanically obstructed bronchus is suspected, bronchoscopy should be performed [22].

Pleural effusion and pulmonary oedema are both conditions characterised by fluid overload, each with a wide range of aetiologies. Pulmonary oedema can be caused neurogenically by aggravated sympathetic stimulation and therefore occur in stroke patients [24]. Both pleural effusion and pulmonary oedema can result from non‐pulmonary conditions such as acute heart failure or acute kidney failure, which are common comorbidities in stroke patients [25, 26, 27]. Loop diuretics as well as pleural tabs should be considered in patients with higher volumes of oedema/effusion. Early treatment may reduce the risk of ARI since fluid overload contributes to a restrictive lung function and therefore increases the risk of ARI [28].

PE emerged as the fifth most prevalent pulmonary condition in our cohort. Several stroke‐related risk factors such as immobility, lower limb paresis, and infections can contribute to the development of deep vein thrombosis and PE [29, 30, 31]. Furthermore, ischaemic stroke as well as PE may even have the same underlying cause, such as antiphospholipid syndrome [32]. Remarkably, one quarter of our cohort presented with PE—patients who might benefit from therapeutic anticoagulation. However, early anticoagulation poses an increased risk of intracranial haemorrhages into necrotic brain parenchyma and must be carefully weighed by physicians [33]. Given the high prevalence of PE, a chest CT‐angiography may be considered liberally in stroke patients with ARI even when they have no additional clinical signs suggestive of PE.

Furthermore, we observed that the majority of patients did not only present with one but with at least three acute pulmonary conditions. The number of conditions proved to be an independent risk factor for patients not being discharged to their homes. Moreover, patients with in‐hospital mortality exhibited changes in vital signs and laboratory parameters indicative of more pronounced ARI, systemic inflammation, and myocardial damage, including increased cardiac Troponin T, PCT, leukocytes, lactate, and heart rate as well as a decreased pH. One potential link between mortality and the observed changes in vital signs might be that the elevated catecholamine levels in stroke patients may trigger a systemic inflammatory response syndrome, which can consequently lead to multiorgan failure and death. In cases of bacterial infections, however, these patients might benefit from early antibiotic treatment, which is a potentially life‐saving therapy and could be further investigated in future clinical trials. It is important to note that clinicians often overestimate the incidence of pneumonia, and that chest x‐rays have lower sensitivity compared to CT scans in accurately diagnosing pneumonia [34]. On that note, our chest CRX findings even reveal that not only pneumonia, but also other common acute pulmonary conditions are frequently underestimated on CRX, thereby reinforcing the rationale for the liberal use of early CT scans. It should be mentioned that a potential selection bias may exist, as the decision to (not) obtain a CXR while carrying out a chest CT scan might have been based on specific clinical circumstances. A previous investigation of chest CT scans of stroke patients at admission identified radiological abnormalities suggestive of aspiration in 15% of patients [35]. These findings align with our study, even though all our patients presented with imaging abnormalities, as we particularly focused on those ARIs due to pulmonary conditions. In both studies, bronchiolitis was the most common radiological finding. The previous study further reported that patients with CT abnormalities had a higher risk of developing clinically overt pneumonia and a greater mortality rate compared to those without such abnormalities [35], thereby supporting our finding that bronchitis/bronchiolitis is associated with increased mortality.

Interestingly, we found that cardiac pre‐morbidities were more prevalent in deceased patients compared to survivors with ARI following a stroke. This finding suggests that the cardiovascular system of deceased patients was more vulnerable prior to admission. It supports the hypothesis that an acute stroke may overwhelm the compensatory capacity of the cardiovascular system, potentially leading to multiorgan failure or cardiac arrest. Furthermore, our study cohort demonstrated a generally high prevalence of cardiovascular risk factors, with arterial hypertension affecting up to 85% (*n* = 187) of all patients. This finding aligns with previous research, which reported a high prevalence (up to 67%) of cardiovascular risk factors in patients with pneumonia post‐stroke who exhibited abnormal chest CT scans [35]. These results indicate that cardiovascular comorbidity may play a relevant role in the development of respiratory failure following a stroke.

Our study has the following limitations: Due to the monocentric and retrospective design, the study cohort represents a local sample that may not be representative of a general stroke cohort, which leads to a low external validity. Our study is therefore considered to be hypothesis‐generating and of exploratory nature. Additionally, we identified patients with ARI by analysing chest CT requests, not by a definition on vital parameters, which introduces another constraint. Nevertheless, the vital signs and laboratory parameters were consistent with an ARI, which validates the approach used for patient selection. Moreover, patients without chest CT scans were not included in our study, as we focused on analysing acute pulmonary alterations using CT imaging. This could potentially lead to a selection bias. Furthermore, in the presence of multiple acute pulmonary conditions, it cannot be concluded which of the acute pulmonary conditions causes an ARI.

## 6 Conclusion

This study explored the pulmonary aetiologies of ARI in ischaemic stroke patients using chest CT scans. In our study cohort, we identified bronchitis/bronchiolitis as well as atelectasis, pleural effusion, pneumonia, pulmonary oedema, and PE as key aetiologies—with bronchitis/bronchiolitis notably linked to in‐hospital mortality. Furthermore, we observed that a higher number of these acute pulmonary conditions was associated with a decreased likelihood of being discharged home. Based on our findings and recent other studies, early chest CT/CT‐angiography may help to identify patients at high risk of mortality or prolonged hospitalisation. Future clinical studies should investigate the benefits of this diagnostic approach, along with preventive and therapeutic measures aimed at improving patient outcomes.

## Author Contributions

**Omid Shirvani:** conceptualization, writing – original draft, methodology, visualization, supervision, project administration, formal analysis, data curation, validation. **Patricia Fischbein:** investigation, writing – original draft, methodology, formal analysis, data curation, conceptualization. **Zeynep Bendella:** writing – review and editing, investigation, methodology. **Piergiorgio Profico:** writing – review and editing, investigation. **Franziska Dorn:** writing – review and editing, resources. **Gabor C. Petzold:** writing – review and editing, resources. **Sebastian Stösser:** conceptualization, writing – review and editing, project administration, supervision, data curation, formal analysis, methodology, validation.

## Ethics Statement

The study was conducted in accordance with the Declaration of Helsinki. The study protocol was approved by the Ethics Committee of the Faculty of Medicine Bonn, BV181/2022.

## Consent

Informed consent was not required from the participants or their legal guardians.

## Conflicts of Interest

The authors declare no conflicts of interest.

## Supporting information


Data S1.

## Data Availability

The raw data is subject to the General Data Protection Regulation of the European Union and can be requested from the corresponding author.
