# Supplementary material for: Aetiology of Acute Respiratory Insufficiency in Patients With Ischaemic Stroke Studied by Chest CT Scan
Source: Eur J Neurol. 2025 Mar 25;32(3):e70125. doi: 10.1111/ene.70125 (PMC11933869; doi:10.1111/ene.70125)
Supplement: Supplementary file 1 — Data S1. [file ENE-32-e70125-s001.pdf]

Supplementary for "Aetiology of acute respiratory insufficiency in patients with ischaemic stroke studied by chest CT scan"

Supplementary Tables

Supplementary Table 1: Clinical data collected from patient medical records.

**Category**	**Variables**
Demographic information	- Age<br>- Sex
Stroke characteristics	- Aetiology (TOAST-Classification)<br>- NIHSS<br>- mRS
Acute stroke treatment	- Intravenous Lysis<br>- Mechanical thrombectomy
Hospitalisation-related data	- Year of treatment<br>- Ward<br>- Length of stay<br>- Discharge mode<br>- Form of diet
Living will	- DNR<br>- DNI
Treatment schedule	- Terminal care decisions
Pre-existing conditions	- Arterial hypertension<br>- Hyperlipoproteinemia<br>- Nicotine abuse<br>- Diabetes mellitus<br>- Pre-existing lung disease<br>- COVID-19<br>- Pre-existing heart disease
Degree of independence	- Barthel index
Vital signs at day of CT scan	- Oxygen saturation level<br>- Respiratory rate<br>- Heart rate<br>- Blood pressure<br>- Temperature<br>- Blood gas analysis<br>- Oxygen administration<br>- Blood test results
Chest CT scan	- Date<br>- Diagnosis<br>- Use of contrast medium

DNI = Do Not Intubate, DNR = Do Not Resuscitate, mRS = Modified Ranking Scale, NIHSS = National Insitutes of Health Stroke Scale, TOAST = Trial of Org 10172 in Acute Stroke Treatment.

Supplementary Table 2: Missing values and data acquisition.

Parameter	Number of missing values	Values acquired by	Method
Age	n = 0	Medical students under supervision of clinical scientists (P.F., P.P., O.S., and S.S.)	Medical records were reviewed
Sex	n = 0		
Arterial hypertension	n = 16		
Diabetes mellitus	n = 27		
Dyslipidaemia	n = 28		
Known cardiac disease	n = 20		
Known pulmonary disease	n = 23		
Active smoker	n = 37		
Premorbid mRS	n = 60		
NIHSS on admission	n = 39		
Intravenous thrombolysis	n = 16		
Endovascular thrombectomy	n = 16		
TOAST	n = 0		
Severity of dysphagia	n = 9		
mRS at discharge	n = 87		
NIHSS at discharge	n = 150		
Exitus letalis during hospitalization	n = 0		
Duration of neurological hospital stay	n = 0		
Discharge mode	n = 0		
Barthel scale	n = 112		
Highest level of care	n = 0		
Pneumonia	n = 0	Board-certified radiologist (Z.B.)	Chest CT scans were reviewed
CT score for pneumonia	n = 0		
Pneumonia with ground glass opacity	n = 0		
Pneumonia with centrilobular nodules	n = 0		

Pneumonia with bronchial wall thickening	n = 0		
Pleural effusion	n = 0		
Volume of pleural effusion	n = 0		
Bronchitis/Bronchiolitis	n = 0		
Atelectasis	n = 0		
Localisation of pulmonary artery embolism	n = 0		
Qanadli Index for pulmonary artery embolism	n = 0		
Type of pulmonary oedema	n = 0		
Respiratory frequency	n = 47	Medical students under supervision of clinical scientists (P.F., P.P., O.S., and S.S.)	Laboratory reports were reviewed
Saturation of oxygen	n = 24		
Heart frequency	n = 18		
Systolic blood pressure	n = 16		
Diastolic blood pressure	n = 16		
Mean arterial blood pressure	n = 16		
Temperature	n = 22		
pH from blood gas analysis	n = 69		
Lactate from blood gas analysis	n = 70		
Leukocytes	n = 29		
C-reactive protein	n = 73		
Cardiac troponin T	n = 82		
Procalcitonin	n = 114		
Signs of oedema	n = 0	Medical student under supervision of clinical scientists (P.F., O.S., and S.S.)	Radiological reports were reviewed
Signs of pleural effusion	n = 0		
Signs of pneumonia	n = 0		
Signs of bronchitis/bronchiolitis	n = 0		
Signs of atelectasis	n = 0		
Signs of pulmonary artery embolism	n = 0		

Signs of other medical conditions	n = 0		

CT = computed tomography, mRS = Modified Ranking Scale, n = number of patients, NIHSS = National Institutes of Health Stroke Scale, TOAST = Trial of Org 10172 in Acute Stroke Treatment. The parameters are listed chronologically according to their order in the tables of the main manuscript.

Supplementary Table 3: Distribution of acute pulmonary diseases diagnosed by CT scan in patients with ischaemic stroke and acute respiratory insufficiency.

**Diagnoses**	**Number of patients - Whole study cohort**	**Number of patients - Survived**	**Number of patients - Deceased**
Bronchitis/Bronchiolitis, n (%)	156 (66.1%)	104 (63.4%)	52 (72.7%)
Atelectasis, n (%)	156 (66.1%)	109 (46.2%)	47 (65.3%)
Pleural effusion, n (%)	143 (60.6%)	98 (41.5%)	45 (62.5%)
Pneumonia, n (%)	125 (53%)	89 (37.7%)	36 (50%)
Pulmonary oedema, n (%)	88 (37.3%)	63 (26.7%)	25 (34.7 %)
Pulmonary artery embolism, n (%)	65 (27.5%)	46 (19.5%)	19 (26.4 %)

n = number of patients.

Supplementary Table 4: Number of acute pulmonary diagnoses per patient.

**Number of acute pulmonary conditions per patient**	**Number of patients**
Six, n (%)	4 (1.7%)
Five, n (%)	45 (19.1%)
Four, n (%)	58 (24.6%)
Three, n (%)	48 (20.3%)
Two, n (%)	40 (17%)
One, n (%)	28 (11.9%)
Zero, n (%)	13 (5.5%)

n = number of patients.

Supplementary Table 5: Logistic regression analysis with pulmonary diagnoses for in-hospital mortality.

**Clinical parameter**	**Beta coefficients (Standard error)**	**Odds ratios (CI)**	**P-values**
Pneumonia	-0.6183 (0.469)	0.5 (0.2, 1.4)	0.19
Bronchitis/Bronchiolitis	1.1536 (0.520)	3.2 (1.1, 8.8)	0.03
Pleural effusion	-0.1330 (0.455)	0.9 (0.4, 2.1)	0.77
Pulmonary artery embolism	-0.3742 (0.423)	0.7 (0.3, 1.6)	0.38
Pulmonary oedema	-0.4461 (0.400)	0.6 (0.3, 1.4)	0.26
Age	0.0101 (0.015)	1.0 (1.0, 1.0)	0.51
Sex	0.2086 (0.373)	1.2 (0.6, 2.6)	0.58
NIHSS at admission	0.1127 (0.032)	1.1 (1.1, 1.2)	<0.01
Intravenous thrombolysis	-0.4994 (0.479)	0.6 (0.2, 1.6)	0.30
Endovascular thrombectomy	0.0978 (0.440)	1.1 (0.5, 2.6)	0.82
Premorbid mRS	0.0688 (0.148)	1.1 (0.8, 1.4)	0.64
Atelectasis	0.1712 (0.451)	1.2 (0.5, 2.9)	0.70

mRS = Modified Ranking Scale, NIHSS = National Institutes of Health Stroke Scale.
95% confidence intervals for coefficients are shown in parentheses.

Supplementary Table 6: Ordinal regression analysis with pulmonary diagnoses for mRS shift.

**Clinical parameter**	**Beta coefficients (Standard error)**	**Odds ratios (CI)**	**P-values**
Pneumonia	-0.0032 (0.405)	1.0 (0.5, 2.2)	0.99
Bronchitis/Bronchiolitis	0.0477 (0.437)	1.0 (0.4, 2.5)	0.91
Pleural effusion	-0.0330 (0.382)	1.0 (0.5, 2.0)	0.93
Pulmonary artery embolism	-0.0081 (0.371)	1.0 (0.5, 2.1)	0.98
Pulmonary oedema	-0.0149 (0.350)	1.0 (0.5, 2.0)	0.97
Age	0.0151 (0.014)	1.0 (1.0, 1.0)	0.26
Sex	0.0055 (0.326)	1.0 (0.5, 1.9)	0.99
NIHSS on admission	0.0890 (0.027)	1.1 (1.0, 1.2)	<0.01
Intravenous thrombolysis	-0.0386 (0.431)	1.0 (0.4, 2.2)	0.93

Endovascular thrombectomy	-0.0183 (0.390)	1.0 (0.5, 2.1)	0.96
Premorbid mRS	-0.0547 (0.124)	0.9 (0.7, 1.2)	0.66
Atelectasis	-0.0001 (0.394)	1.0 (0.5, 2.2)	1.00

mRS = Modified Ranking Scale, NIHSS = National Institutes of Health Stroke Scale.
95% confidence intervals for coefficients are shown in parentheses.

Supplementary Table 7: Ordinal regression analysis with pulmonary diagnoses count for mRS shift.

**Clinical parameter**	**Beta coefficients (Standard error)**	**Odds ratios (CI)**	**P-values**
Pulmonary diagnoses count	-0.0434 (0.109)	1.0 (0.8, 1.2)	0.69
Age	0.0351 (0.013)	1.0 (1.0, 1.1)	0.01
Sex	-0.0895 (0.321)	0.9 (0.5, 1.7)	0.78
NIHSS on admission	0.0866 (0.027)	1.1 (1.0, 1.1)	0.00
Intravenous thrombolysis	-0.0293 (0.409)	1.0 (0.4, 2.2)	0.94
Endovascular thrombectomy	0.0398 (0.382)	1.0 (0.5, 2.2)	0.92
Premorbid mRS	0.0285 (0.122)	1.0 (0.8, 1.3)	0.82

mRS = Modified Ranking Scale, NIHSS = National Institutes of Health Stroke Scale.
95% confidence intervals for coefficients are shown in parentheses.

Supplementary Table 8: Logistic regression analysis with pulmonary diagnoses count for in-hospital mortality.

**Clinical parameter**	**Beta coefficients (Standard error)**	**Odds ratios (CI)**	**P-values**
Pulmonary diagnoses count	0.0710 (0.121)	1.1 (0.8, 1.4)	0.56
Age	0.0100 (0.015)	1.0 (1.0, 1.0)	0.50
Sex	0.1571 (0.358)	1.2 (0.6, 2.4)	0.66
NIHSS on admission	0.1049 (0.030)	1.1 (1.0, 1.2)	0.00
Intravenous thrombolysis	-0.5367 (0.460)	0.6 (0.2, 1.4)	0.24

Endovascular thrombectomy	-0.0755 (0.419)	0.9 (0.4, 2.1)	0.86
Premorbid mRS	0.0728 (0.143)	1.1 (0.8, 1.4)	0.61

mRS = Modified Ranking Scale, NIHSS = National Institutes of Health Stroke Scale.
95% confidence intervals for coefficients are shown in parentheses.

Supplementary Table 9: Linear regression analysis with pulmonary diagnoses for duration of neurological hospital stay.

**Clinical parameter**	**Beta coefficients (Standard error)**	**P-values**
Pneumonia	2.8 (2.5)	0.27
Bronchitis/Bronchiolitis	-1.0 (2.7)	0.71
Pleural effusion	-1.5 (2.3)	0.52
Pulmonary artery embolism	-1.2 (2.2)	0.58
Pulmonary oedema	-2.2 (2.1)	0.30
Age	-0.2 (0.1)	0.02
Sex	-1.4 (2.0)	0.49
NIHSS on admission	-0.0 (0.2)	0.79
Intravenous thrombolysis	1.5 (2.6)	0.55
Endovascular thrombectomy	1.1 (2.3)	0.65
Premorbid mRS	-1.0 (0.8)	0.22
Atelectasis	3.8 (2.3)	0.10

mRS = Modified Ranking Scale, NIHSS = National Institutes of Health Stroke Scale.
95% confidence intervals for coefficients are shown in parentheses.

Supplementary Table 10: Linear regression analysis with pulmonary diagnoses count for duration of neurological hospital stay.

**Clinical parameter**	**Beta coefficients (Standard error)**	**P-values**
Pulmonary diagnoses count	0.3 (0.7)	0.65
Age	-0.2 (0.1)	0.01
Sex	-1.8 (2.0)	0.38
NIHSS on admission	-0.1 (0.2)	0.74
Intravenous thrombolysis	1.3 (2.6)	0.62
Endovascular thrombectomy	0.9 (2.3)	0.69

Premorbid mRS	-1.1 (0.8)	0.16

mRS = Modified Ranking Scale, NIHSS = National Institutes of Health Stroke Scale.
95% confidence intervals for coefficients are shown in parentheses.

Supplementary Table 11: Logistic regression analysis with pulmonary diagnoses for discharge at home.

**Clinical parameter**	**Beta coefficients (Standard error)**	**Odds ratios (CI)**	**P-values**
Pneumonia	-0.2384 (0.592)	0.8 (0.2, 2.5)	0.69
Bronchitis/Bronchiolitis	-1.0828 (0.596)	0.3 (0.1, 1.1)	0.07
Pleural effusion	-0.3280 (0.497)	0.7 (0.3, 1.9)	0.51
Pulmonary artery embolism	-0.2639 (0.521)	0.8 (0.3, 2.1)	0.61
Pulmonary oedema	-0.0305 (0.508)	1.0 (0.4, 2.6)	0.95
Age	-0.0006 (0.017)	1.0 (1.0, 1.0)	0.97
Sex	-0.7226 (0.475)	0.5 (0.2, 1.2)	0.13
NIHSS on admission	-0.1390 (0.043)	0.9 (0.8, 0.9)	0.00
Intravenous thrombolysis	0.8681 (0.537)	2.4 (0.8, 6.8)	0.11
Endovascular thrombectomy	-0.2902 (0.624)	0.7 (0.2, 2.5)	0.64
Premorbid mRS	0.0966 (0.175)	1.1 (0.8, 1.6)	0.58
Atelectasis	-0.8810 (0.483)	0.4 (0.2, 1.1)	0.07

mRS = Modified Ranking Scale, NIHSS = National Institutes of Health Stroke Scale.
95% confidence intervals for coefficients are shown in parentheses.

Supplementary Table 12: Logistic regression analysis with pulmonary diagnoses count for discharge at home.

**Clinical parameter**	**Beta coefficients (Standard error)**	**Odds ratios (CI)**	**P-values**
Pulmonary diagnoses count	-0.5810 (0.157)	0.6 (0.4, 0.8)	<0.01
Age	0.0104 (0.017)	1.0 (1.0, 1.0)	0.54
Sex	-0.7274 (0.484)	0.5 (0.2, 1.2)	0.13
NIHSS on admission	-0.1027 (0.045)	0.9 (0.8, 1.0)	0.02
Intravenous thrombolysis	0.8537 (0.566)	2.3 (0.8, 7.1)	0.13

Endovascular thrombectomy	-0.3790 (0.659)	0.7 (0.2, 2.5)	0.57
Premorbid mRS	0.1064 (0.177)	1.1 (0.8, 1.6)	0.55

mRS = Modified Ranking Scale, NIHSS = National Institutes of Health Stroke Scale.
95% confidence intervals for coefficients are shown in parentheses.

Supplementary Figures

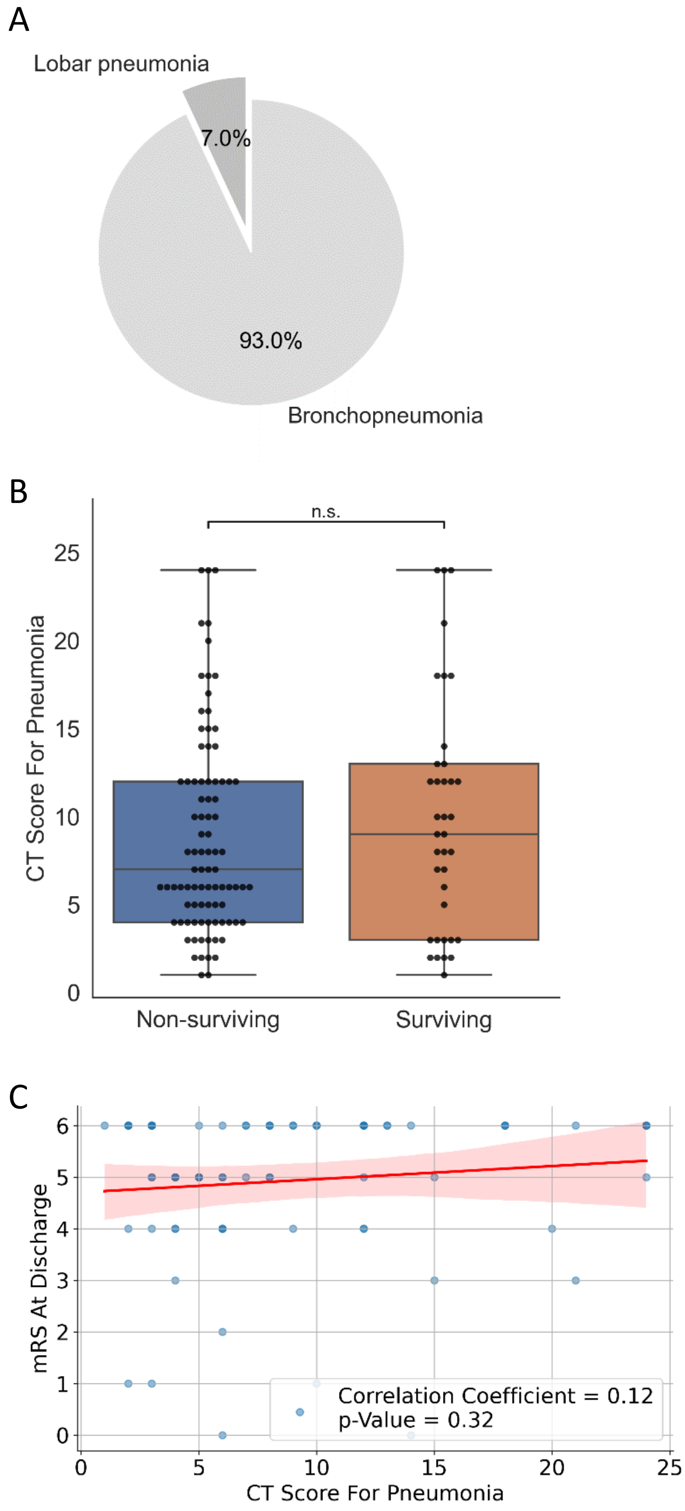

Supplementary Figure 1: A) Type of pneumonia among patients with alveolar or mixed (alveolar + interstitial) pneumonia. B) CT score between surviving and non-surviving patients. C) Spearman correlation between CT score for pneumonia and modified Ranking Scale (mRS) at discharge.

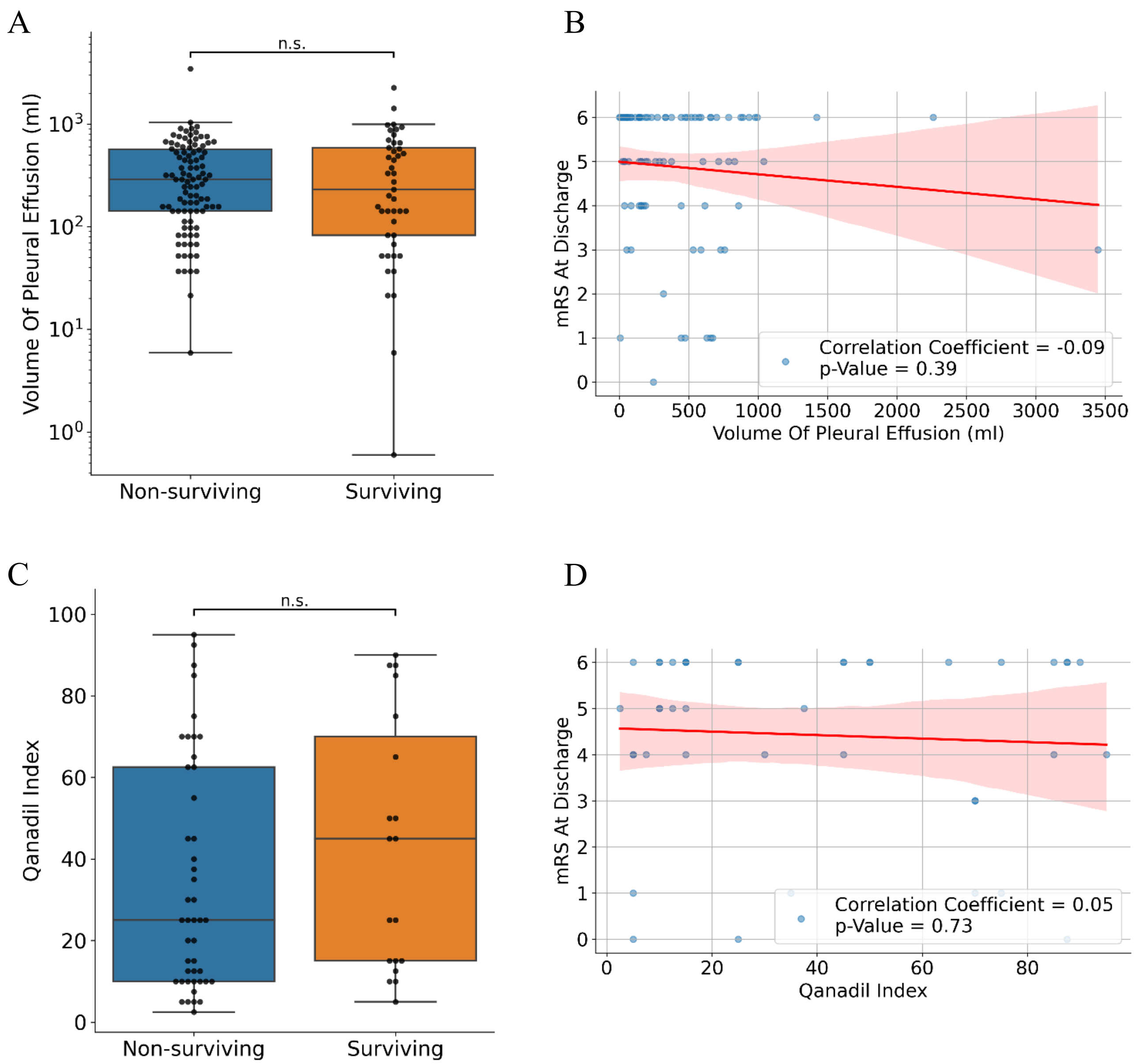

Supplementary Figure 2: A) Volume of pleural effusion between surviving and non-surviving patients. B) Spearman correlation between volume of pleural effusion and modified Ranking Scale (mRS) at discharge. C) Qanadli Index between surviving and non-surviving patients. D) Spearman correlation between Qanadli Index and mRS at discharge.
